# Chromosomal instability and molecular mutations in multi spectrum disease of Fanconi anemia

**DOI:** 10.1186/1755-8166-7-S1-I47

**Published:** 2014-01-21

**Authors:** Babu Rao Vundinti

**Affiliations:** 1 ICMR- National Institute of Immunohaematology, Parel, Mumbai, India

## 

Fanconi anemia (FA; MIM no. 227650), the most commonly inherited bone marrow disorder, has an overall prevalence of 1–5 per million and an estimated carrier frequency of 1:200-300 in most populations. Demonstrating either an autosomal or X-linked recessive mode of inheritance, FA is characterized by childhood progressive bone marrow failure and predisposition to acute myelogenous leukemia, older patients are at increased risk for squamous cell carcinomas of the head, neck, and genitourinary tract. Congenital abnormalities are present in approximately 70% of FA patients and may include radial ray defects, cafe´ au lait spots or hypopigmentation, short stature, microphthalmia, malformations of kidneys, gastrointestinal tract, and heart, mental retardation, and hearing defects. Because of the high degree of phenotypic variability exhibited by FA patients, diagnosis may be difficult on the basis of clinical manifestations alone. Because FA patient-derived lymphocytes and fibroblasts exhibit hypersensitivity to DNA crosslinking agents such as diepoxybutane (DEB) and mitomycin C (MMC), resulting in a high rate of chromosomal breakage and radial formation, analysis on the basis of this hypersensitivity has been routinely used to confirm clinical diagnosis.

Molecular diagnosis of FA has been challenging because of the genetic heterogeneity associated with the disease. To date, 15 FA gene products (FANCA, B, C, D1, D2, E, F, G, I, J, L, M, N, O and P) have been identified and they constitute the FANC pathway, which is thought to function in preventing genome instability. The FA core complex comprises FAAP24, FAAP100, and 8 FA proteins (FANCA, B, C, E, F, G, L, and M) and mediates DNA-damage-induced or replication-stress induced monoubiquitylation of FANCD2 and FANCI. Monoubiquitinated FANCD2 and FANCI translocate to chromatin and function in DNA repair at least partially by recruitment of FAN1 nuclease. Cloning of the *FANC* genes has provided a means, via complementation group analysis, to routinely pinpoint the patient’s specific FA gene harboring the mutations, thus greatly simplifying subsequent molecular analysis. Although retroviral-mediated complementation analysis of FA patient-derived cells is commonly used to identify the patient’s genetic subtype as a prerequisite for mutation screening, such analysis has not been formally validated for clinical use. Previous studies have considered Fanconi complementation Group A (FA-A) assignments, but none have performed comprehensive sequencing to verify complementation assignment and/or evaluated complementation assignments performed using retroviral-mediated rescue as evidenced by correction of MMC and DEB-induced chromosome breakage and radial formation. Among all complementation groups, FAA accounts for the majority of FA patients (60–65%) followed by FAC (10%) and FAG (9%). Mutations in the gene for complementation group FA-A (*FANCA*; MIM no. 607139) confer autosomal recessive inheritance and account for approximately 66% of all FA cases. The *FANCA* gene is mapped to chromosome 16q24.3,11 spans approximately 80 kb of genomic DNA (gDNA), and consists of 43 exons. With more than 200 different mutations described thus far, the *FANCA* mutation spectrum is very heterogeneous. Because of the presence of a large number of *Alu* repeat sequences within the *FANCA* gene, large intragenic deletions involving multiple exons have been reported to account for more than 40% of all *FANCA* mutations.

First time we have carried out a molecular study in the Indian population. We have studied FA by chromosomal breakage study using mitomycin C (MMC) and diepoxybutane (DEB) induction and FANCD2 monoubiquitination by western blotting to understand gene defects in FA pathway. The complementation analysis was done by retroviral transfection. The molecular study was carried out by Multiplex Ligation-dependent Probe Amplification (MLPA) and direct sequencing of FANCA, C, G, E, F, L, and M genes. The complementation analysis results showed different spectrum as compared to world literature. Our study revealed FA-A and E gene defects in 69% cases and followed by FANCE gene defect. The molecular analysis showed the large deletions in 11 patients and FANCA gene mutations were in 12 FA patients. Out of 12 mutations 5 mutations (c.3678 C>G, p.Ser 1226 X, c.3993G>A p.Leu 1331 Pro, c.1274 C>G p.Glu 425 His, c.2630 C>G p.Ser 877 X) found to be novel mutations. In our series the single nucleotide polymorphisms (SNP); Exon9,c.796A>G(p.Thr266Ala), Exon16,c.1501G>A(p.Gly501Ser), Exon26, c.2426G>A(p.Gly809Asp), of FANCA gene also observed in 23 patients, and these polymorphisms in disease association was reported in FA database, however, it needs to be established in Indian population. Interesting finding of our study is the existence of FANCE mutation in Indian population, and the same was reported rarely in other places of the world. The study also highlighted the uncharacterized FA patients, which may associated with new genes in Indian population and these patients should be studied molecularly and genotype-phenotype correlation need to be established, which helps in better understanding and management of the disease.

